# Exposure to (*Z*)-11-hexadecenal [(*Z*)-11-16:Ald] increases *Brassica nigra* susceptibility to subsequent herbivory

**DOI:** 10.1038/s41598-021-93052-8

**Published:** 2021-06-29

**Authors:** Agnès Brosset, Monirul Islam, Sara Bonzano, Massimo E. Maffei, James D. Blande

**Affiliations:** 1 Department of Environmental and Biological Sciences, University of Eastern Finland, Yliopistonranta 1 E, N70211 Kuopio, Finland; 2 Plant Physiology Unit, Department of Life Sciences and Systems Biology, University of Turin, Via Quarello 15/A, 10135 Turin, Italy; 3 Neuroscience Institute Cavalieri Ottolenghi (NICO) Regione Gonzole, 10 - 10043 Orbassano (TO), Italy; 4 Present Address: Department of Sustainable Crop Production, Università Cattolica Del Sacro Cuore, Via Emilia Parmense 84, 29122 Piacenza, Italy

**Keywords:** Chemical ecology, Ecophysiology, Plant signalling, Plant stress responses, Plant sciences

## Abstract

It is well established that plants emit, detect and respond to volatile organic compounds; however, knowledge on the ability of plants to detect and respond to volatiles emitted by non-plant organisms is limited. Recent studies indicated that plants detect insect-emitted volatiles that induce defence responses; however, the mechanisms underlying this detection and defence priming is unknown. Therefore, we explored if exposure to a main component of *Plutella xylostella* female sex pheromone namely (*Z*)-11-hexadecenal [(*Z*)-11-16:Ald] induced detectable early and late stage defence-related plant responses in *Brassica nigra.* Exposure to biologically relevant levels of vapourised (*Z*)-11-16:Ald released from a loaded septum induced a change in volatile emissions of receiver plants after herbivore attack and increased the leaf area consumed by *P. xylostella* larvae. Further experiments examining the effects of the (*Z*)-11-16:Ald on several stages of plant defence-related responses showed that exposure to 100 ppm of (*Z*)-11-16:Ald in liquid state induced depolarisation of the transmembrane potential (Vm), an increase in cytosolic calcium concentration [Ca^2+^]_cyt_, production of H_2_O_2_ and an increase in expression of reactive oxygen species (ROS)-mediated genes and ROS-scavenging enzyme activity. The results suggest that exposure to volatile (Z)-11-16:Ald increases the susceptibility of *B. nigra* to subsequent herbivory. This unexpected finding, suggest alternative ecological effects of detecting insect pheromone to those reported earlier. Experiments conducted in vitro showed that high doses of (*Z*)-11-16:Ald induced defence-related responses, but further experiments should assess how specific the response is to this particular aldehyde.

## Introduction

Studies since the early 1980s have increased our knowledge on the biology and ecology of plant responses to plant-emitted volatile organic compounds (VOCs)^1^. Accumulating evidence has shown that VOCs also participate in between- and within plant-signalling^2–4^ structuring the coordination of defences within a plant, and providing cues to plants of the same and different species^5,6^. Through these cues, plants can obtain information about their environment, and optimize their performance by altering their growth in response to indicators of competition^7^ or prime their defences in response to a threat^8,9^. However, our knowledge on the ability of plants to detect and respond to volatile cues from non-plant organisms has received much less attention^10^.

In 2004, a study showed that the volatile blends of two bacteria, *Bacillus subtilis* and *Bacillus amyloliquefaciens*, could induce systemic resistance in plants and reduce the severity of the disease caused by the pathogen *Erwinia carotovora* subsp. *carotovora*^11^. They identified 2,3-butanediol as the compound in the volatile blend that induced resistance. Helms and colleagues^12–14^ showed for the first time that plants exposed to volatiles emitted by insects can prime their defences in response. Their work showed that goldenrod *Solidago altissima* plants exposed to the male sex pheromone of the goldenrod gall fly *Eurosta solidaginis* had higher concentrations of the defence-related phytohormone jasmonic acid in their leaves and emitted a greater amount of herbivore-induced plant volatiles (HIPVs) than unexposed plants^12,13^. In subsequent experiments they identified that (*E*,*S*)-conophthorin, the most abundant compound in the sex pheromone of the goldenrod gall fly, was responsible for priming goldenrod defences^14^. They also reported that exposure to the pheromone reduced the performance of the specialist herbivore *Trirhabda virgata* on goldenrod plants, but not on *Cucurbita pepo* or *Symphyotrichum lateriflorum* plants, suggesting that the defence priming was restricted to a plant–insect combination with a co-evolutionary relationship^13^. A recent study on *Pinus sylvestris* showed that pine sawfly (*Diprion pini*) pheromone primed defences to be stronger upon the early stages of damage by sawfly larvae. The authors also showed that exposure to the pheromone increased hydrogen peroxide (H_2_O_2_) concentrations in pine needles, as well as expression of defence-related pine genes leading to a reduction of the survival of subsequently laid sawfly eggs^15^.

*Plutella xylostella* L., is a widely distributed moth and specialist pest of Brassicaceous plants. A few hours after emergence, females release a sex pheromone that acts as short to long-distance signals to attract conspecific males^16,17^. Several hours after mating, females lay eggs on *Brassicaceae* foliage and stem, and a couple of days later eggs hatch into first instar larvae that start consuming the mesophyll of the leaves. (*Z*)-11-hexadecenal [(*Z*)-11-16:Ald)] is one of the major components of the sex pheromone emitted by *P. xylostella* females^18^. In the present study, we addressed the following questions (1) does (*Z*)-11-16:Ald induce detectable defence-related responses in *Brassica nigra* plants when applied as a volatile chemical to the air surrounding a plant? (2) does exposure to (*Z*)-11-16:Ald elicit early and late defence-related responses in vitro and (3) is the response specific to the (*Z*)-11-16:Ald component of the sex pheromone? To address question 1, we sampled the volatile emissions of plants after exposure to (*Z*)-11-16:Ald, and after subsequent feeding by first and second instar *P. xylostella* larvae, and the amount of feeding by the larvae after 24 h. To address question 2 we measured a selection of the early and late signalling events following exposure to (*Z*)-11-16:Ald, including the plasma membrane potential (Vm), cytosolic calcium flux, production of hydrogen peroxide, (ROS)-mediated genes and ROS-scavenging enzyme activity. We addressed question 3 by measuring the Vm of plants exposed to (*Z*)-11-hexadecenyl acetate ((*Z*)-11-16:Ac) another component of the sex pheromone.

We hypothesized that (*Z*)-11-16:Ald would prime plant defences leading to a change in volatile emissions after exposure and a decrease of feeding by *P. xylostella* larvae. We also hypothesized that the priming would be specific to (*Z*)-11-16:Ald and lead to an activation of early and late defence signalling events.

## Results

### Experiment 1: Effects of (*Z*)-11-16:Ald on *Brassica nigra* volatile emissions and feeding by *Plutella xylostella* larvae

**Figure 1:**
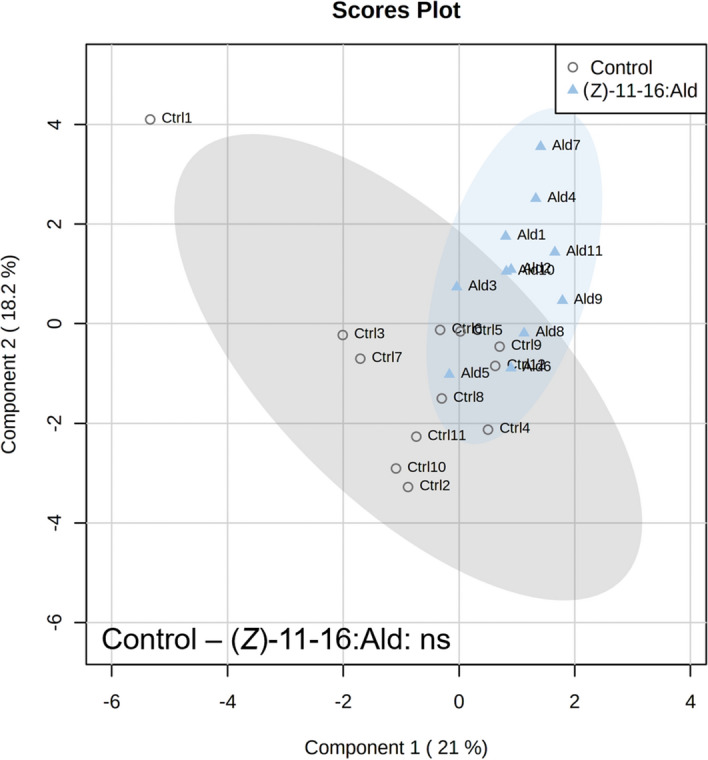
Partial Least Squares – Discriminant Analysis (PLS-DA) based on plant volatile emissions after plant exposure to vapourised (*Z*)-11-16:Ald and non-exposed controls with cross validation based on 50 submodels (fivefold outer loop and fourfold inner loop). Pairwise tests were performed, based on PLS-DA with 999 permutations, to highlight the difference between treatments with n = 9 to 12. ns indicates non-significant differences.

**Figure 2:**
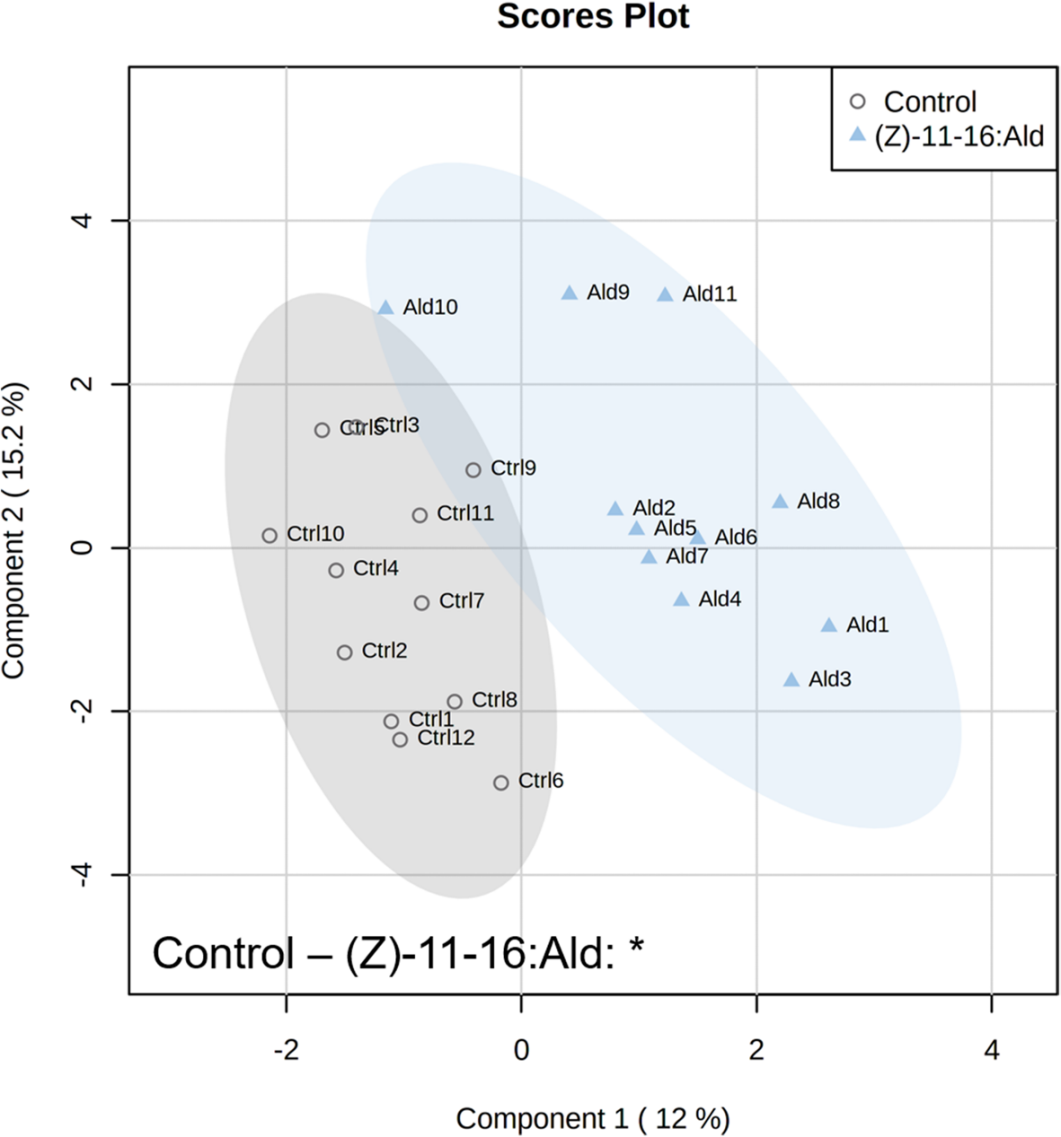
Partial Least Squares – Discriminant Analysis (PLS-DA) based on plant volatile emissions after 24 h of feeding by *Plutella xylostella* with cross validation based on 50 submodels (fivefold outer loop and fourfold inner loop). Pairwise tests were performed, based on PLS-DA with 999 permutations, to highlight the difference between treatments with n = 9 to 12. * *P* < 0.05.

**Figure 3:**
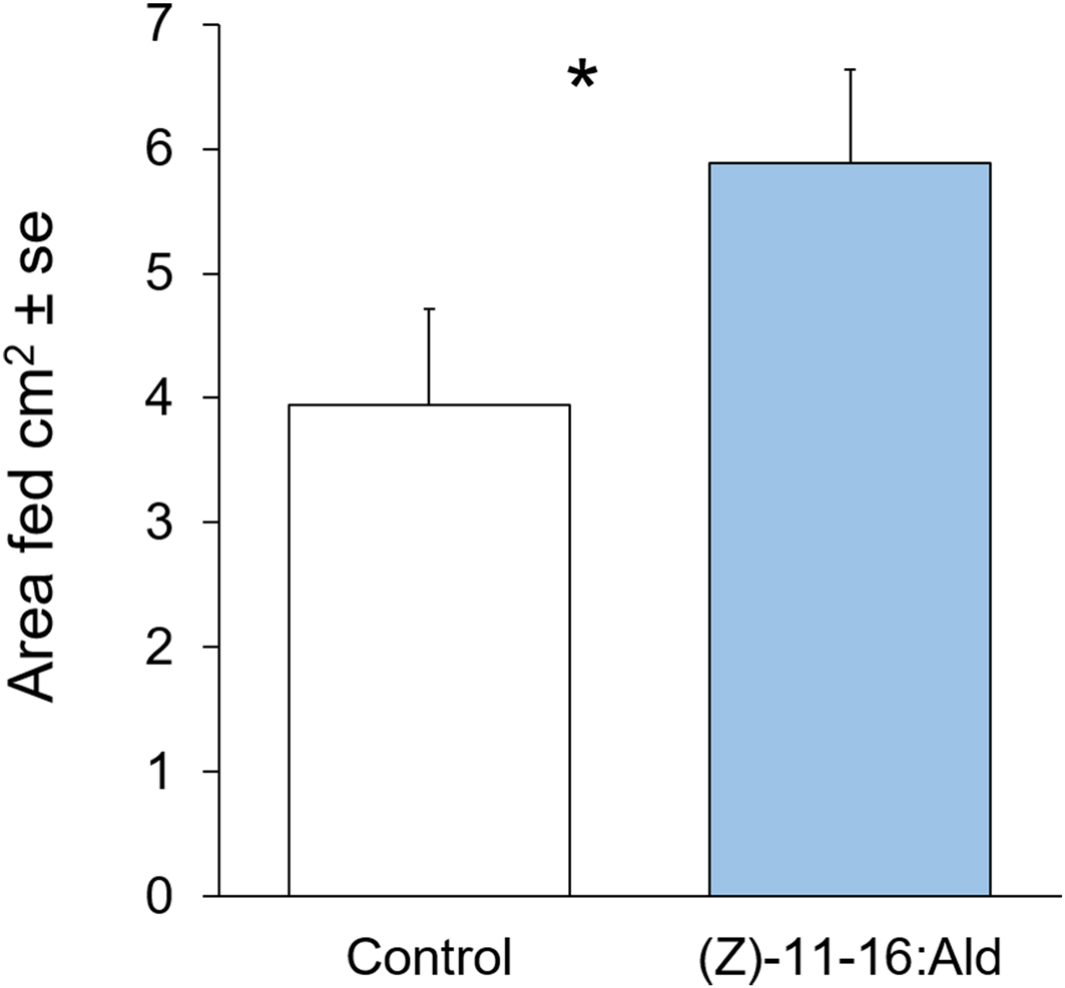
Amount of *Brassica nigra* leaf consumed by *Plutella xylostella* larvae in 24 h after exposure of plants to septa loaded with 100 µl of a 100 ppm (*Z*)-11-16:Ald solution vapourised over 24 h (n = 10), or non-exposed controls (n = 12). Error bars indicate SE, * *P* < 0.05.

To test whether (*Z*)-11-16:Ald induces detectable defence-related responses in *B. nigra* plants, treated plants were exposed for 24 h to (*Z*)-11-16:Ald released from septa loaded with 100 µl of a 100 ppm (*Z*)-11-16:Ald solution. Volatile emissions were collected from both control and (*Z*)-11-16:Ald-exposed plants before and after *P. xylostella* larvae feeding on the plants for 24 h. Immediately after exposure, volatile emissions did not differ between the control and (*Z*)-11-16:Ald-exposed plants (*P* = 0.507) (Fig. 1). However, after 24 h of feeding by *P. xylostella* larvae, the volatile emissions were significantly different (*P* = 0.039) (Fig. 2). At the end of the feeding time, and after the collection of VOC, we measured the area consumed by larvae and found that *P. xylostella* larvae fed more on plants exposed to (*Z*)-11-16:Ald than plants exposed to clean air (*P* = 0.044) (Fig. 3).

### Experiment 2: Effects of *Plutella xylostella* pheromone components on the transmembrane potential of *Brassica nigra* plants

**Figure 4:**
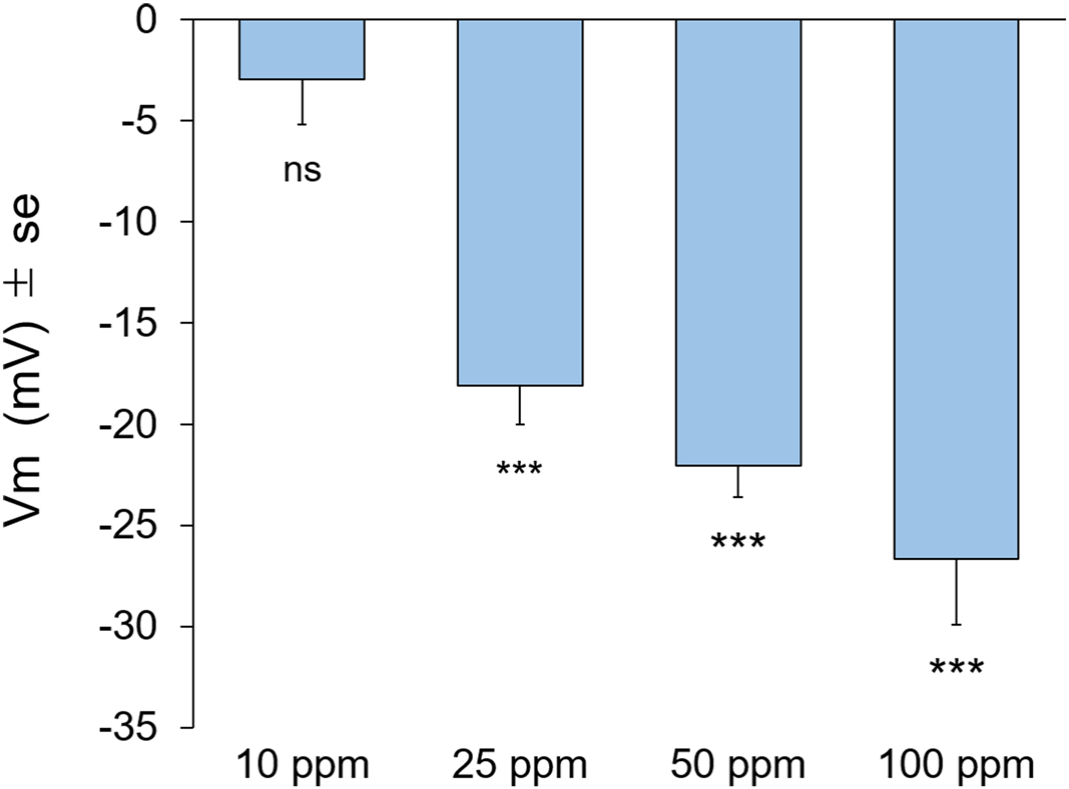
Membrane potential (Vm) depolarisation of plants after perfusion of leaf segments with (*Z*)-11-16:Ald. Leaves were perfused for 1 h with a solution containing MES buffer + 0.1% Tween 20 and different concentrations of (*Z*)-11-16:Ald. Vm depolarisation indicates the difference in membrane potential between the mean values of each treatment with the control being a solution of MES buffer + 0.1% Tween 20. Perfusion with the (*Z*)-11-16:Ald induced a Vm depolarisation of *Brassica nigra* cells. Error bars indicate SE. *** *P* < 0.001. ns indicates non-significant differences. n = 3 to 4 for each treatment.

A change in transmembrane potential (Vm) is known to be the first step in plant detection of chemical cues^19^. We consequently studied whether the insect-emitted compounds could lead to a change in Vm of *B. nigra* leaves by applying 10 to 100 ppm aqueous solutions directly to leaf segments. Treatment with (*Z*)-11-16:Ald elicited Vm depolarisation at all concentrations tested except 10 ppm suggesting a threshold between 25 and 10 ppm (Fig. 4). In this experiment we also tested the effects of (*Z*)-11-16:Ac, another component of *P. xylostella* sex pheromone, on the transmembrane potential of *B. nigra*. Treatment with (*Z*)-11-16:Ac only elicited Vm depolarisation at 100 ppm (Table S1) showing that plants are more sensitive to (*Z*)-11-16:Ald exposure.

### Experiment 3: Effect of (*Z*)-11-16:Ald on intracellular calcium concentrations and H_2_O_2_ production

**Figure 5:**
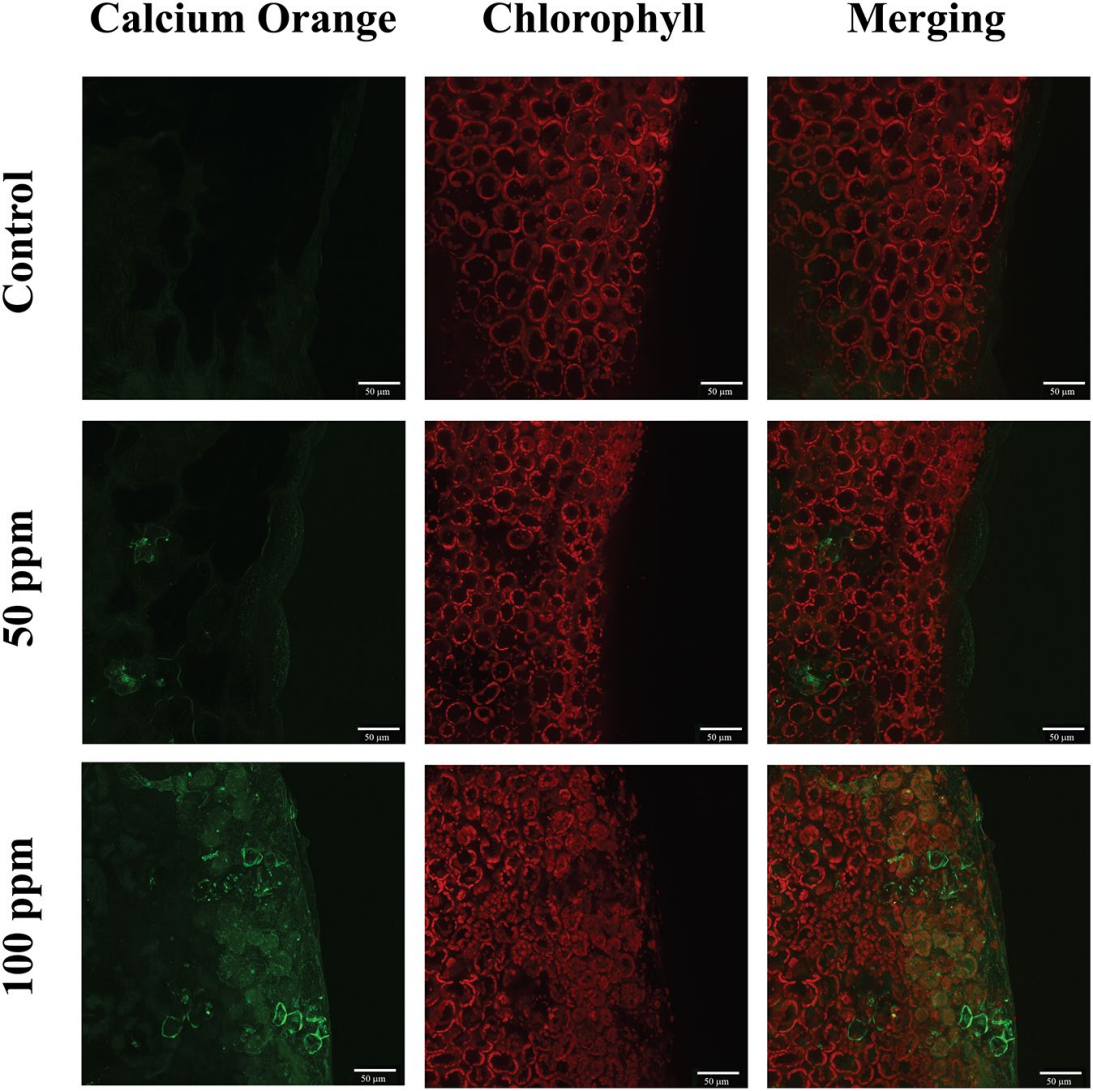
False-colour image analysis reconstructions from confocal laser scanning microscope observations and fluochemical intracellular Ca^2+^ determination. The green fluorescence indicates the presence of Ca^2+^ in cells, whereas the chloroplasts are shown by a bright red colour caused by chlorophyll fluorescence. Leaves of *Brassica nigra* were incubated for 20 min with MES buffer + 0.1% Tween 20 as control, and either 50 ppm or 100 ppm of (*Z*)-11-16:Ald (diluted in MES buffer + 0.1% Tween 20). Scale bars are indicated on pictures. Exposure to (*Z*)-11-16:Ald increased [Ca^2+^] in cells exposed to the molecule. n = 2 for each treatment.

**Figure 6:**
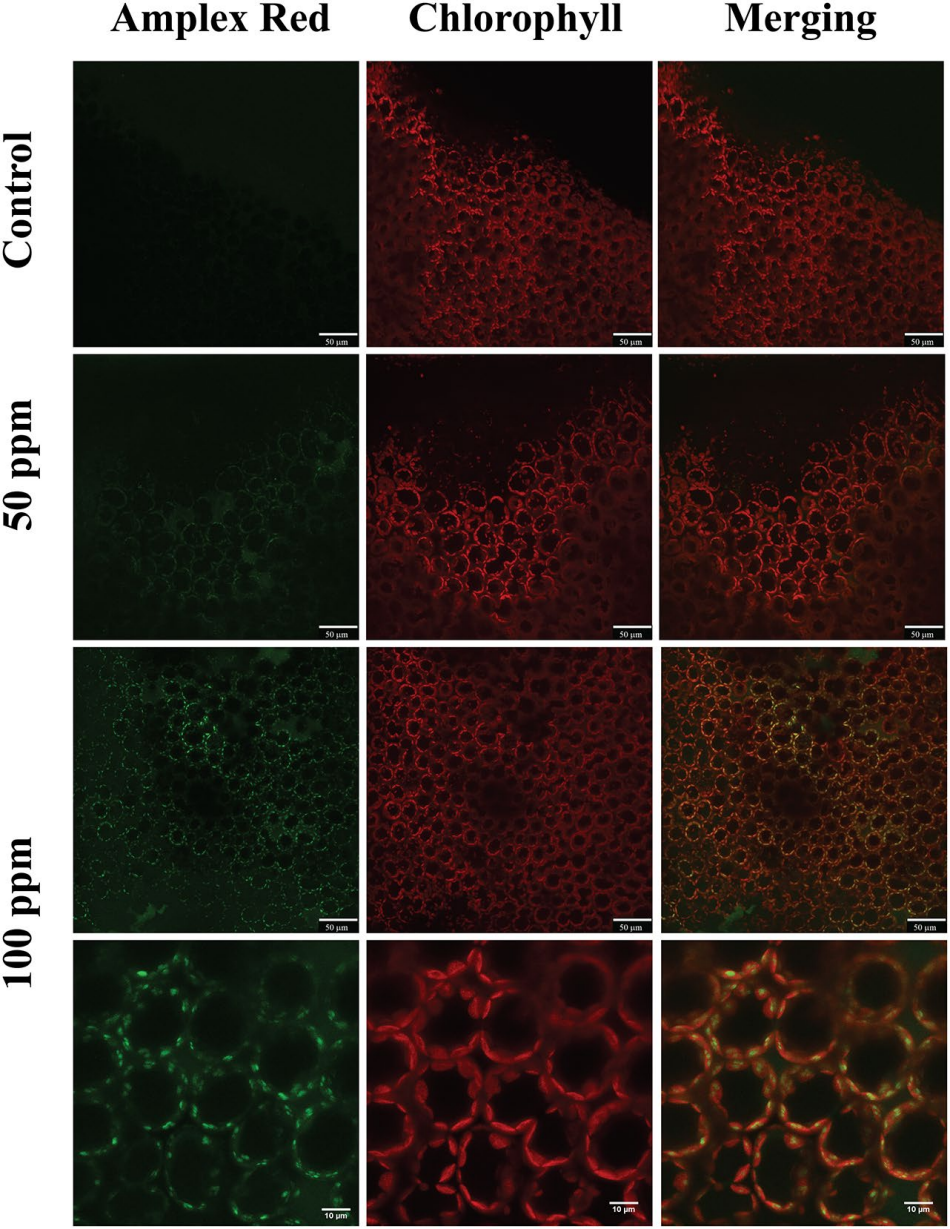
False-colour image analysis reconstructions from confocal laser-scanning microscope observations and fluochemical localization of H_2_O_2_ in an image analysis. The production of H_2_O_2_ is shown by the green fluorescence. The chloroplasts are shown by a bright red colour caused by chlorophyll fluorescence. *Brassica nigra* leaves were incubated for 20 min with MES buffer + 0.1% Tween 20 as control, and 50 ppm or 100 ppm of (*Z*)-11-16:Ald (diluted in MES buffer + 0.1% Tween 20). Scale bars are indicated on pictures. Exposure to (*Z*)-11-16:Ald at 100 ppm leads to accumulation of H_2_O_2_ in cells. n = 2 for each treatment.

Vm variations are associated with variations in cytosolic calcium concentration ([Ca^2+^]_cyt_)^20,21^. Therefore, we determined [Ca^2+^]_cyt_ in plant leaves after 30 min of incubation with 50 and 100 ppm of (*Z*)-11-16:Ald in aqueous solution (Fig. 5). Exposure to both concentrations increased intracellular [Ca^2+^], but the strongest green fluorescence was observed after exposure to 100 ppm of (*Z*)-11-16:Ald. Since an increased [Ca^2+^]_cyt_ is often associated with an increased production of ROS^22^, we proceeded to determine H_2_O_2_ production in plant leaves and observed that plants treated with 100 ppm of (*Z*)-11-16:Ald, showed a strong production of H_2_O_2_ in treated cells, with respect to controls (Fig. 6).

### Experiment 4: The effect of (*Z*)-11-16:Ald on ROS-scavenging enzyme activities and gene expression

**Figure 7:**
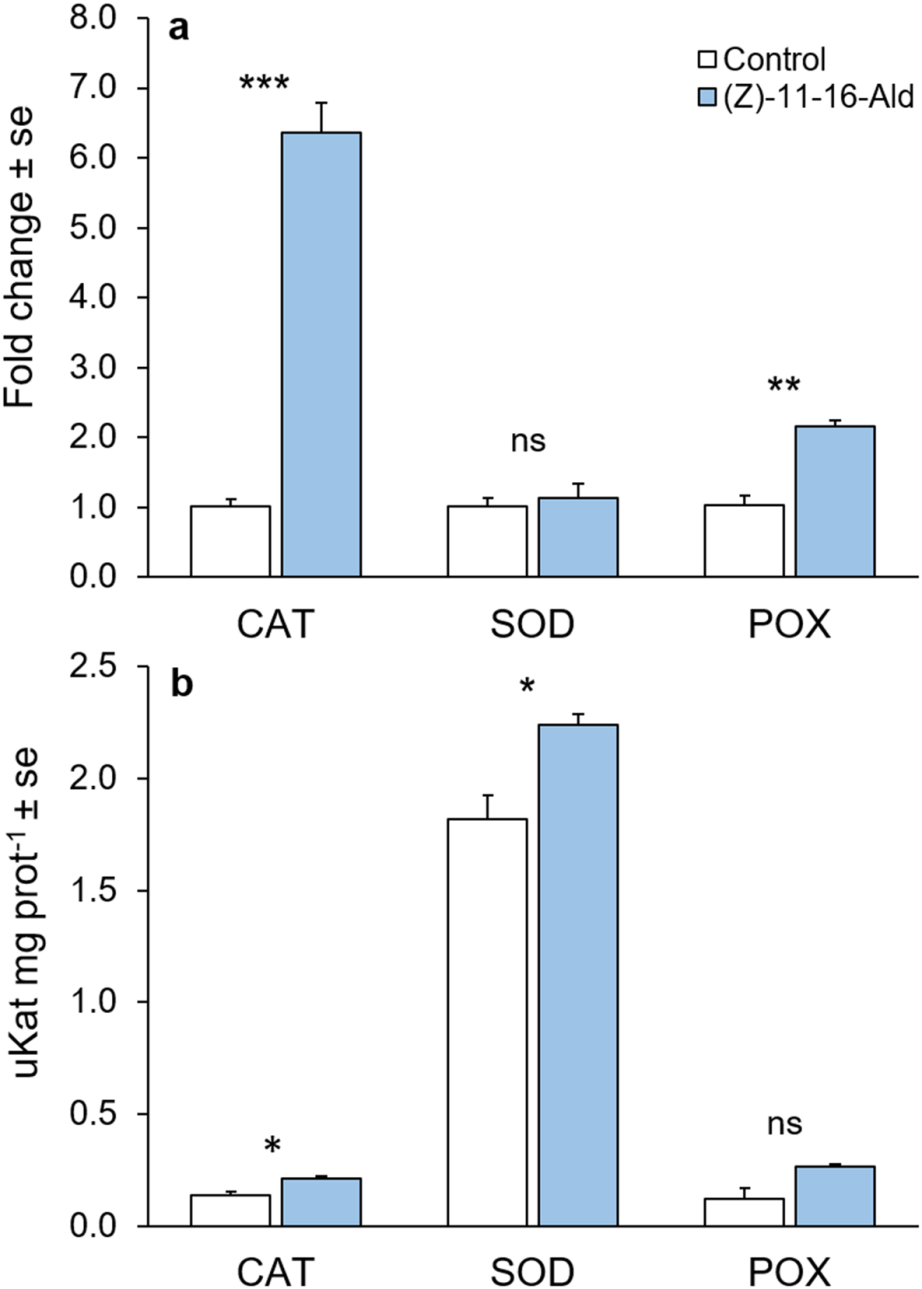
Gene expression (**a**) and enzyme activity (**b**) of three ROS-scavenging enzymes. Quantitative gene expression and enzyme activity were assessed after 30 min of exposure of plants to 100 ppm of (*Z*)-11-16:Ald in solution of MES buffer + 0.1% Tween 20 or exposure to a solution of MES buffer + 0.1% Tween 20 as control. Error bars indicate se. * *P* < 0.05, ** *P* < 0.01, *** *P* < 0.001. ns indicates non-significant. n = 3 to 4 for each treatment.

In order to assess whether the ROS production observed with the confocal laser scanning microscope (CLSM) was associated with increased enzyme activity and transcription, we measured the activities of ROS scavenging enzymes catalase (CAT), superoxide dismutase (SOD), and peroxidase (POX) and their associated gene expression for control plants and plants treated for 30 min with 100 ppm of (*Z*)-11-16:Ald in aqueous solution. CAT gene transcript levels and enzyme activity were both significantly (*P* < 0.001, Fig. 7a, and *P* = 0.007, Fig. 7b) enhanced by 100 ppm (Z)-11-16:Ald treatment. SOD enzyme activity increased in response to (*Z*)-11-16:Ald treatment (*P* = 0.011, Fig. 7b), but there was no detectable change at the transcriptomic level (*P* = 0.647, Fig. 7a). For POX, (*Z*)-11-16:Ald increased the gene transcript level (*P* = 0.002, Fig. 7a) and tended to increase the enzyme activity (*P* = 0.056, Fig. 7b).

## Discussion

To determine whether exposure to (*Z*)-11-16:Ald induced detectable defence-related responses in *B. nigra*, we investigated the amount of feeding damage to plants and the volatile emissions of plants exposed to volatilised pure compound. Contrary to our hypothesis, *P. xylostella* larvae fed more on plants previously exposed to (*Z*)-11-16:Ald at biologically relevant levels than on controls, suggesting that exposure to (*Z*)-11-16:Ald might increase the susceptibility of plants to future herbivory. Earlier work investigating the responses of plants to insect sex pheromone showed that exposure primed defences^14,16^, resulting in higher volatile emissions^13^, and lower feeding^12^. Our results are different to these findings and suggest alternative ecological effects of detecting insect pheromone to those reported earlier.

The greater feeding on (*Z*)-11-16:Ald-exposed plants compared to controls could relate to the differences in volatile emissions of the differently treated plants. Although there were no detectable differences in volatile emissions between exposed plants and controls immediately after (*Z)*-11-16:Ald-exposure, there were differences after a subsequent 24 h of feeding. The difference could be due to (*Z*)-11-16:Ald-induced changes in the plant directly affecting herbivore-induced volatile emission, or could be related to altered plant nutrition or defences leading to an increase in the leaf area consumed by herbivores and a consequent difference in induction of volatile emissions. Thus (*Z*)-11-16:Ald could potentially alter plant defences and in doing so increase the survival of eggs and future hatched larvae. It has earlier been shown in *Arabidopsis thaliana* that application of *Pieris brassicae* or *Spodoptera littoralis* egg extract onto leaves reduced the induction of genes related to defence against insects after caterpillar feeding^23^. However, at this stage our data does not allow us to further explore these ecological hypotheses. Additional studies would need to test in greater detail the deposition of eggs, the hatching of eggs, and performance and survival of developing larvae of plants exposed to (*Z*)-11-16:Ald.

The observation of changes in feeding amount and plant volatile responses prompted us to examine the early and late signaling events in response to (*Z*)-11-16:Ald exposure. The results showed that exposure to (*Z*)-11-16:Ald induced a transmembrane depolarisation by plants. The depolarisation of the plasma membrane potential is known to be the first detectable event in the detection of a biotic or abiotic stress^19^. We estimated the detection threshold of the pheromone to be a concentration between 25 and 10 ppm. A lower level of transmembrane depolarisation was observed when plants were exposed to (*Z*)-11-16:Ac, hence (*Z*)-11-16:Ald appears to be the most phytoactive component of the *P. xylostella* sex pheromone. However, it remains unclear how specific the plant response is to the (*Z*)-11-16:Ald, which should be further elucidated by comparison with aldehydes that have a more similar chemical structures.

The plasma membrane is the only cellular structure in direct contact with the environment, which makes it critical for sensing environmental stimuli and initiating a cascade of events that eventually leads to a specific response^21^. As demonstrated in *Arabidopsis*, Vm depolarisation depends on a cascade of events that include changes in [Ca^2+^]_cyt_ and the production of ROS^24^. We found that 50 ppm and 100 ppm of (*Z*)-11-16:Ald increased both the [Ca^2+^]_cyt_ and the production of ROS. Moreover, we demonstrated that the increased ROS detected by CLSM were associated with the increased expression of reactive oxygen species (ROS)-mediated genes and ROS-scavenging enzyme activity. ROS participate in cell oxidation, during which H_2_O_2_ is produced and later regulated by ROS-scavenging enzymes involved in its degradation to protect cells from oxidative stress^25,26^. The production of H_2_O_2_ is potentially harmful and can result in oxidation in the cells of aerobic living organisms^27^. It is also an important component of the signalling network in plants^28,29^ and takes part in plant defence in response to environmental stress^30,31^. Several plant species trigger localized cell death by producing ROS at oviposition sites on leaves, which has been shown to be associated with an increase in egg mortality or a reduction of larval survival rate^32^. Recently, Bittner and colleagues demonstrated that when plants are previously exposed to the female pine sawfly (*D. pini*) sex pheromone, they produce H_2_O_2_ and induce defence-related genes faster after egg deposition on leaves, compared to plants that have not been exposed to the pheromone^15^. They suggested that the sex pheromone acted as an environmental cue indicating to plants that there would be future egg deposition on needles and subsequent herbivory. Plants then responded to it by producing H_2_O_2_, which formed necrotic tissue and reduced survival of eggs. Taken together with our observations, it is possible that the (*Z*)-11-16:Ald also primes *B. nigra* plant defences by producing H_2_O_2_ as a defensive mechanism to limit future egg deposition. It was shown that *B. nigra* plants induce the necrosis of cells located at the oviposition site of *Pieris rapae* and *Pieris napi*^33^*,* which can support this hypothesis*.* Contrary to the hypothesis of defence induction, *B. nigra* plants exposed to (*Z*)-11-16:Ald received more herbivore-feeding damage than control plants, but further investigations are needed to determine whether the production of H_2_O_2_ following exposure to (*Z*)-11-16:Ald would reduce subsequent egg deposition or hatching.

Interestingly, early signalling events following the exposure to (*Z*)-11-16:Ald are analogous to the responses induced by biotic stress such as herbivore-wounding^20,22^, and in response to HIPVs^21^. For example, exposure to HIPVs resulted in a depolarisation of the plasma membrane (Vm)^34,35^ due to the entrance of calcium (Ca^2+^) into the cytosol of cells^34^. The detection of HIPVs results in transcriptional^36,37^, metabolic and physiological changes in plants^38^. Several studies have shown that plants perceiving HIPVs deploy faster and stronger chemical defences upon subsequent stress^8,9^, which can negatively affect herbivorous insects^39^. The observed action of (*Z*)-11-16:Ald is typical to that of insect and mite elicitors^21,40,41^. However, it is notable that to determine if (*Z*)-11-16:Ald induced detectable responses in *B. nigra* plants, whole plants were exposed to vapourised (*Z*)-11-16:Ald for 24 h, while to determine early and late signalling events following exposure to (*Z*)-11-16:Ald we applied 100 ppm in aqueous state for 30 min to 1 h (Table S2). While we used a biologically realistic scenario with a realistic concentration of pheromone for the whole plant responses the in vitro experiments focussed on mechanisms do not represent biologically accurate scenarios and utilised high concentrations of pheromone. Future studies should bridge this methodological gap by utilising more biologically realistic scenarios in mechanism elucidation.

(*Z*)-11-16:Ald has been found to be a main constituent of pheromones in many moth species from the Noctuidae family including the corn earworm *Helicoverpa zea*^42^, which is the second-most important economic pest species in North America^43^. Many plants that have co-evolved with the Noctuidae could have the ability to detect (*Z*)-11-16:Ald. Prior to this study, the ability of plants to detect insect-emitted volatiles had been reported for two species: a perennial plant, *S. altissima*, and a tree, *P. sylvestris.* We can now tentatively add an annual plant, *B. nigra*, to the list. These studies suggest that the ability to detect insect-emitted volatiles has widely evolved in a large variety of plant families, and highlight the need to determine how widespread this trait is. Further studies should also determine the threshold and distance of detection and the ecological consequences of this detection.

In summary our results indicate for the first time that exposing *B. nigra* plants to volatile (*Z*)-11-16:Ald increases the susceptibility of plants to feeding by *P. xylostella* larvae and induces an alteration in herbivore-induced volatile emissions. Further mechanistic experiments conducted in vitro using high doses of pheromone indicated that exposure to (*Z*)-11-16:Ald induces responses in receiver plants that are characterised by a depolarisation of Vm, an increase in [Ca^2+^]_cyt_ and production of H_2_O_2_ leading to an increase in ROS-mediated gene expression and ROS scavenging-enzyme activity, which are typical responses to insect elicitors. This study supports recent findings showing that plants can detect insect-emitted volatiles. However, further research should be conducted to determine an accurate dose response of whole plants to volatile pheromone and the specificity of the response to this particular aldehyde.

### Materials and methods

The study complied with local and national regulations.

### Plants

*Brassica nigra* (black mustard) seeds were collected from wild populations in the Netherlands (supplied by E. Poelman, Wageningen University). For experiment 1, conducted in Kuopio (Finland), plants were grown in plastic pots (8 × 8 cm) containing a mix of peat, soil and sand (3:1:1), in plant growth chambers (Weiss Bio 1300 m, Germany) with a 16L: 8D and light intensity of 250 µmol m^−2^ s^−1^. The temperature was maintained at 21 °C with 60% relative humidity during the day and decreased to 16 °C with 80% RH during the night time. Plants were watered every day and fertilized twice per week with a 0.1% solution containing nitrogen, phosphorous and potassium in a 19:4:20 ratio (Kekkilä Oyj, Finland).

For experiments conducted in Turin (Italy) (Experiments 2 to 4), plants were grown in plastic pots (8 × 8 cm) containing a mix of peat, soil (Klasmann-Deilmann, Germany), sand (Vimark, Italy) and vermiculite (Unistara, Italy), in a climate-controlled room at 22 ± 1 °C, 16L:8D with light intensity of 250 µmol m^−2^ s^−1^. Three and four-week-old plants were used for all the experiments.

### Insects and synthetic compounds

*Plutella xylostella* were reared on broccoli (*B. oleracea* var. *italica*) with an artificial light–dark cycle of 16L:8D at 22 ± 0.5 °C.

Synthetic (*Z*)-11-hexadecenal [(*Z*)-11-16:Ald] and (*Z*)-11-hexadecenyl acetate [(*Z*)-11-16:Ac] (isomeric purity 93%), were purchased from Pherobank (Wageningen, The Netherlands).

### Exposure of plants to treatments

A 100 ppm solution of synthetic (*Z*)-11-16:Ald diluted in dichloromethane was prepared and 100 µl of the solution was injected into a rubber septum (7 mm O.D; Sigma-Aldrich) and left for 30 min for the dichloromethane to evaporate. As a solvent control, 100 µl of dichloromethane was deposited on a rubber septum, and left to evaporate for 30 min. A second control, without the rubber septum and dichloromethane, was also set up. Either treatment or control septa were enclosed in 0.5 L glass jars connected with Teflon tubing to plastic bags (Polyethylene terephthalate; overall dimensions 28 × 35 cm; Look Isopussi Eskimo oy, Finland). Plants were enclosed in the PET bags, that were previously baked for 1 h at 120 °C. For 24 h, a clean air flow was passed into the treatment or control glass jars and then into the bags containing the plants (Fig. S1 and Table S2).

### Volatile collections and feeding assays

After 24 h of exposure to the treatments, jars containing the rubber septa were disconnected from the plants, and a first VOC sampling was made before adding 22 first and second instar *P. xylostella* larvae were added to each plant for 24 h. After 24 h of feeding, volatile compounds were collected by dynamic headspace sampling (Fig. S1). VOCs were trapped by pulling clean air at 0.22 L min^−1^ for one hour through steel tubes filled with 200 mg Tenax TA 60/80 adsorbent (Markes International Ltd, UK) using a vacuum pump (KNF, Germany). The collected volatiles were thermally desorbed and analysed by gas chromatography-mass spectrometry (Agilent 7890A GC, and Agilent MS model 5975C VL MSD; New York, USA). Trapped compounds were desorbed with a thermal desorption unit (TD-100; Markes International Ltd, Llantrisant, UK) at 250 °C for 10 min, and cryofocused at − 10 °C in splitless mode onto an HP‐5 capillary column (50 m, 0.2 mm i.d, 0.5 μm film thickness; Hewlett‐Packard). The oven temperature programme was held at 40 °C for 1 min and then ramped 5 °C min^−1^ to 210 °C, and then further ramped at 20 °C min^−1^ to 290 °C. The carrier gas was helium, the transfer line temperature to the MSD was 300 °C, the ionization energy was 70 eV, and the full scan range was 29–355 m/z. We identified volatiles by comparison with a series of analytical standards (Sigma-Aldrich, Germany) and by comparison of their mass spectra to those in the NIST and Wiley 275 mass spectral libraries. Compound quantification was based on Total Ion Chromatograms (TIC) and according to the responses of analytical standards. Emission rates (ER) were calculated with the formula ER = X*Ai/Dw*t*Ao. ER was expressed in ng gDW^−1^ h^−1^, X was the compound quantity (ng), Ai and Ao were the inlet and outlet air flows (mL min-1), respectively, t was the sampling time of one hour and Dw is the dry weight of the plant sampled (g).

After the volatile collection, we placed leaves of plants on A4 paper for scaling and digitally photographed them. Plants were then dried in paper bags in an oven at 60 °C for 3 days. The leaf area consumed by larvae was calculated using the LeafAreaAnalyzer software (https://github.com/EmilStalvinge/LeafAreaAnalyser; emilstalvinge@gmail.com).

### Transmembrane potential (Vm) measurements

Leaf segments (0.5 cm^2^) of three individual *B. nigra* plants were placed in Eppendorf tubes for 1 h with either 1 ml of (*Z*)-11-16:Ald solution, (*Z*)-11-16:Ac solution or control (Table S2). Vm was determined by inserting an electrode into a plant leaf segment following a method detailed earlier^44^. Vm variations were measured upon perfusion of four concentrations: 10, 25, 50, and 100 ppm diluted in MES buffer (pH 6.5) + 0.1% Tween 20 (V/V). A solution of MES buffer (pH 6.5) + 0.1% Tween 20 (V/V) was used as control. Vm values were recorded every five seconds.

### Determination of intracellular calcium variations using confocal laser scanning microscopy (CLSM) and Calcium Orange

Calcium Orange dye (stock solution in DMSO, Molecular Probes, Leiden, The Netherlands) was diluted in 5 mM MES-Na buffer (pH 6.0) to a final concentration of 5 µM. This solution was applied to *B. nigra* leaves attached to the plant as previously reported^20,24,45^. After 1 h incubation with Calcium Orange, the leaf was mounted on a Leica TCS SP2 (Leica Microsystems Srl, Milan, Italy) multiband confocal laser scanning microscope (CLSM) stage, without separating the leaf from the plant, in order to assess the basic fluorescence levels as control. A 50 µl application of either 50 or 100 ppm (*Z*)-11-16:Ald was made and after 30 min the calcium signature was observed. The microscope operates with a Krypton/Argon laser at 543 nm and 568 nm wavelengths: the first wavelength excites Calcium Orange, resulting in green fluorescence and the second mainly excites chlorophyll, resulting in red fluorescence. All images were obtained with an objective HCX APO 40 × immersed in water with a numeric aperture (NA) of 0.8. The scan speed was set at 400 Hz (Hz). The microscope pinhole was set at 0.064 mm and the average size depth of images was between 65 and 70 µm; the average number of sections per image was 25. The image format was 1024 × 1024 pixels, 8 bits per sample and 1 sample per pixel.

### CLSM Subcellular localization of H_2_O_2_ and active peroxidases using 10-acetyl-3,7-dihydroxyphenoxazine (Amplex Red)

*B. nigra* leaves from rooted potted plants were treated with 50 µl of either 50 or 100 ppm of (*Z*)-11-16:Ald (Table S2) after incubation with the dye 10-acetyl-3,7-dihydroxyphenoxazine (Amplex Red) as described earlier^22^. The Molecular Probes Amplex Red Hydrogen Peroxide/Peroxidase Assay kit (A-22188) was used and dissolved in MES-Na buffer 50 mM (pH6.0) containing 0.5 mM calcium sulfate to obtain a 50 μM solution. Leaves where then mounted on a Leica TCS SP2 miscroscope as described above. Scannings were recorded after 180 min using the HCX PL APO 63x/1.20 W Corr/0.17CS objective. The microscope was operated with a Laser Ar (458 nm/5 mW; 476 nm/5 mW; 488 nm/20 mW; 514 nm/20 mW); a Laser HeNe 543 nm/1,2 mW and a Laser HeNe 633 nm/10 mW.

### ROS-scavenging enzyme activities and soluble protein determination

Leaves were collected immediately after 30 min of exposure to either 100 ppm of (*Z*)-11-16:Ald or control (Table S2). Intact leaves of two pooled plants were frozen in liquid N_2_ and stored at -80ºC before enzyme extraction. Frozen leaves were used for extraction of ROS scavenger enzymes following the method described in Maffei et al.^22^. All steps were carried out at 4 °C. Plant material was ground with a mortar and pestle under liquid nitrogen in cold 50 mM sodium phosphate buffer, pH 7.5, containing 250 mM sucrose, 1.0 mM EDTA, 10 mM KCl, 1 mM MgCl_2_, 0.5 mM phenylmethylsulfonyl fluoride (PMSF), 0.1 mM dithiothreitol (DTT), and 1% (w/v) polyvinylpolypyrrolidone (PVPP) in a 1:10 proportion (weight of plant material to buffer volume). The homogenate was then centrifuged at 25,000 g for 20 min at 4 °C and the supernatant was used directly for measurement of enzyme activity. The soluble protein concentration was measured using the method established by Bradford^46^ using bovine serum albumin as a standard.

Catalase (CAT) activity was assayed spectrophotometrically by monitoring the absorbance change at 240 nm due to the decreased absorption of H_2_O_2_ (ɛ = 39.4 mM^−1^ cm^−1^). The reaction mixture in 1 mL final volume contained 50 mM Na-P, pH 7.0, 15 mM H_2_O_2_, and the enzyme extract. The reaction was initiated by addition of H_2_O_2_.

Peroxidase (POX) activity was measured by detecting the oxidation of guaiacol (ɛ = 26.6 mM^−1^ cm^−1^) in the presence of H_2_O_2_. The reaction mixture contained 50 mM Na-P, pH 7.0, 0.33 mM guaiacol, 0.27 mM H_2_O_2_, and the enzyme extract in a 1.0 mL final volume. The reaction was started by addition of guaiacol and measured spectrophotometrically at 470 nm.

Superoxide dismutase (SOD) activity was measured by reduction of nitro blue tetrazolium due to a photochemically generated superoxide anion. One ml of assay mixture consisted of 50 mM Na-P buffer, pH 7.8, 13 mM methionine, 75 μM nitro blue tetrazolium (NBT), 2 μM riboflavin, 0.1 mM EDTA, and the enzyme extract. Riboflavin was added as the last reagent. Samples were placed 30 cm below a light source (60 µmol m^−1^ s^−1^), and the reaction was allowed to run for 15 min. The reaction was stopped by switching off the light. A non irradiated reaction mixture, which was run in parallel, did not develop colour and served as a control. The absorbance was read at 560 nm.

### Quantitative gene expression analysis by Real-time PCR

Total RNA was isolated from control or treatment leaf tissues using RNA Isolation mini Kit (Machery-Nagel, Germany), and RNase- Free DNase according to the manufacturer’s protocols. The quality of RNA was checked in 1% agarose gel and the final yield was checked with a Spectrophotometer (Pharmacia Biotech Ultrospec 3000, United States). The cDNA synthesis was performed starting from 1 µg RNA using the High Capacity cDNA Reverse Transcription kit (Applied Biosystem, United States). Primers for real-time PCR were designed using the Primer 3.0 software^47^ and the relative sequences are listed in Supplementary Table S3. The real-time PCR was performed on an Mx3000P Real-Time System (Agilent Technologies, United States) using SYBR green I with the dye ROX as an internal loading standard. The reaction mixture was 10 µl in volume and comprised 5 µl of 2 × Maxima SYBR Green qPCR Master Mix (Thermo Fisher Scientific), 0.5 ml of cDNA, and 100 nM of primers (Integrated DNA Technologies, United States). The thermal conditions were as follows: 10 min at 95 °C, 40 cycles of 15 s at 95 °C, 20 s at 57 °C, and 30 s at 72 °C. Fluorescence was read after each annealing and extension phase. All runs were followed by a melting curve analysis from 55 to 95 °C. Two reference genes, *ACT1 and* eEF1Balpha2, were used to normalize the results. The sequences of the primers used in this work for *CAT1*, *CuZnSOD1, PER4, ACT1* and *eEF1Balpha2* are reported in Table S3. All amplification plots were analyzed with the MX3000P software (Agilent Technologies, United States) to obtain Ct values. Real-time PCR data are expressed as fold change of the treatment with respect to the control.

### Statistical analysis

Area consumed by larvae, Vm measurements, enzyme activity and gene expression data were analysed using SPSS 25 software (IBM Corp. Armonk, USA). The normality of data and homogeneity of variances were checked and log transformed when the data did not meet assumptions for parametric analyses. Because we observed no significant differences between the control and the solvent control, we directly compared the control with (*Z*)-11-16:Ald for all analyses. Differences between treatments were analysed using T-tests for the fed area, gene expression and enzyme activity. Volatile emission rates were log transformed and auto-scaled (mean-centered and divided by the standard deviation of each variable). Partial Least Squares – Discriminant Analysis (PLS-DA) was performed on emission rates with the R software (v. 3.4.3) with the package vegan and RVAideMemoire with cross validation based on 50 submodels (fivefold outer loop and fourfold inner loop). A pairwise test was performed, based on PLS-DA with 999 permutations, to highlight the differences between treatments. The PLS-DA graphics were done with metaboanalyst (https://www.metaboanalyst.ca/).

## Supplementary Information

Supplementary Information.

## Data Availability

The datasets generated during the current study are available from the corresponding author on reasonable request.

## Abbreviations

VOC: Volatile organic compounds
HIPV: Herbivore-induced plant volatiles
H_2_O_2_: Hydrogen peroxide
ROS: Reactive oxygen species
Vm: Transmembrane potential
[Ca^2^^+^]_cyt_: Cytosolic calcium concentration
CLMS: Confocal laser scanning microscrope
CAT: Catalase
ppm: Parts-per-million
POX: Peroxidase
SOD: Superoxide dismutase
(*Z*)-11-16Ald: (*Z*)-11-Hexadecenal
(Z)-11-16Ac: (Z)-11-hexadecenyl acetate
