# Supplementary material for: Exposure to (Z)-11-hexadecenal [(Z)-11-16:Ald] increases Brassica nigra susceptibility to subsequent herbivory
Source: Sci Rep. 2021 Jun 29;11:13532. doi: 10.1038/s41598-021-93052-8 (PMC8242006; doi:10.1038/s41598-021-93052-8)
Supplement: Supplementary file 1 — Supplementary Information. [file 41598_2021_93052_MOESM1_ESM.docx]

**Exposure to (*Z*)-11-hexadecenal [(*Z*)-11-16:Ald] increases *Brassica nigra* susceptibility to subsequent herbivory.**

Agnès Brosset^1*^, Monirul Islam^2^, Sara Bonzano^2,3^, Massimo E. Maffei^2^, James D. Blande^1^

^1^Department of Environmental and Biological Sciences, University of Eastern Finland, Yliopistonranta 1 E, Kuopio FIN-70211, Finland

^2^ Plant Physiology Unit, Department of Life Sciences and Systems Biology, University of Turin, Via Quarello 15/A, 10135 Turin, Italy

^3^Neuroscience Institute Cavalieri Ottolenghi (NICO) Regione Gonzole, 10 - 10043 Orbassano (TO), Italy

* Corresponding author E-mail: [agnes.brosset@uef.fi](mailto:agnes.brosset@uef.fi)

**Table S1**Data represent the mean difference between the control Vm and the mean for each treatment and the standard errors. Leaves were exposed for 1h in a solution containing MES buffer + 0.1% ethanol and different concentrations of (*Z*)-11-hexadecenal and (*Z*)-11-hexadecenyl acetate individually. The control is a solution of MES buffer + 0.1% ethanol.

| Treatment | Concentration (ppm) | Mean ± se | p.value |
| --- | --- | --- | --- |
| (*Z*)-11-16:Ald | 10 | -2.96 ± 1.51 | ns |
| (*Z*)-11-16:Ald | 25 | -18.09 ± 1.00 | ******* |
| (*Z*)-11-16:Ald | 50 | -22.05 ± 2.57 | ******* |
| (*Z*)-11-16:Ald | 100 | -26.63 ± 3.25 | ******* |
| (*Z*)-11-16:Ac | 10 | 4.65 ± 1.43 | ns |
| (*Z*)-11-16:Ac | 25 | -8.39 ± 1.10 | ns |
| (*Z*)-11-16:Ac | 50 | -8.91 ± 1.88 | ns |
| (*Z*)-11-16:Ac | 100 | -12.17 ± 1.56 | ****** |

**Table S2** Concentrations of (*Z*)-11-16:Ald, area of plant tissue exposed to (*Z*)-11-16:Ald and time of exposure tested for each experiment. VOC: Volatile organic compounds, Vm: transmembrane potential, [Ca^2+^]_cyt_: cytosolic calcium concentration, H_2_O_2_: hydrogen peroxide, and ppm: parts-per-million.

| Experiment | Concentration tested | Area of plant tissue exposed | Exposure time |
| --- | --- | --- | --- |
| Experiment 1: VOC and feeding essays | 100ppm (vapor) | Whole plant | over 24h |
| Experiment 2: Vm measurements | 10, 25, 50 and100ppm | 0.5cm^2^ of leaf | 1h |
| Experiment 3: [Ca^2+^]_cyt_ and H_2_O_2_ production | 50 and 100ppm | Whole leaf | 30min |
| Experiment 4: enzyme activities and protein determination | 100ppm | Whole leaf | 30min |

**Table S3**List of primers used in this work.

| **Gene Code** | **Gene** | **Forward primer (5’-3’)** | **Reverse primer (5’-3’)** |  |
| --- | --- | --- | --- | --- |
| **REFERENCE GENES** | | | | |
| At2g37620 | *ACT1* | TGCACTTCCACATGCTATCC | GAGCTGGTTTTGGCTGTCTC |  |
| At1g30230 | *eEF1Balpha2* | GCACCATTGTTGACGACCTT | GGCAACAATGTCACAGCTCT |  |
| **TARGET GENES** | | | | |
| At1g20630 | *CAT1* | CAGGACTACAGGCACATGGA | CGACAGGCATTTGATCCCAC |  |
| At1g08830 | *SOD1* | CTGGTCCACATTTCAACCCC | ATTGTGAAGGTGGCAGTTCC |  |
| At1g14540 | *PER4* | TAGAGAGTGTTTGTCCCGGC | GATAGCCCTAAACGCGTTGG |  |

**Fig. S1** Experimental design for testing the volatile responses of *Brassica nigra* plants exposed to (*Z*)-11-16:Ald. Clean air flow (a) was channelled from a glass jar containing the rubber septum(b) to the plastic bags enclosing plants (c). After 24h, the rubber septum was removed from the system, volatile organic compounds were collected by headspace sampling into Stainless steel Tenax TA-filled tubes (d) using a vacuum pump (e). *Plutella larvae* were added to the plants (c) to fed for 24h before re-collecting volatile organic compounds.

**Table S4** Mean (±SE) emission rates (ng g^−1^ h^−1^) of volatile compounds emitted by *Brassica nigra* exposed to vapourised 100ppm (*Z*)-11-16:Ald over a course of 24h (n=11), dichloromethane as a solvent control (n=9) or non-exposed controls (n=12), before larval feeding and after 24h of feeding by *Plutella xylostella* larvae. Differences in emissions rates were analysed using Kruskal Wallis tests. NA indicates that no statistical test was performed. Letters indicate significant differences between treatments. (*E*)-DMNT: (*E*)-4,8-Dimethylnona-1,3,7-triene.

|  | t=0 | | |  | t=24 | | |  |
| --- | --- | --- | --- | --- | --- | --- | --- | --- |
|  | Control | Solvent | (*Z*)-11-16:Ald | *P*.value | *Control* | *Solvent* | (*Z*)-11-16:Ald | *P*.value |
| Butanol | 60.17 ± 52.01 | 10.91 ± 4.64 | 12.53 ± 3.22 | 0.568 | 38.82 ± 26.32 | 8.36 ± 4.16 | 11.87 ± 3.48 | 0.134 |
| 3-Butenenitrile | 0.00 ± 0.00 | 0.00 ± 0.00 | 0.00 ± 0.00 | 0.435 | 6.22 ± 4.12 | 0.13 ± 0.13 | 0.00 ± 0.00 | 0.196 |
| Dimethyldisulfide | 16.52 ± 8.23 | 4.78 ± 2.36 | 2.67 ± 0.89 | 0.123 | 16.8 ± 10.10 | 7.00 ± 4.02 | 1.98 ± 1.01 | 0.261 |
| *cis*-3-hexenol | 0.47 ± 0.47 | 0.00 ± 0.00 | 3.72 ± 3.72 | 0.662 | 0.36 ± 0.36 | 2.32 ± 2.32 | 3.59 ± 2.56 | 0.724 |
| Allyl-isothiocyanate | 5.27 ± 3.84 | 4.61 ± 4.08 | 4.73 ± 4.56 | 0.968 | 75.88 ± 24.87 | 74.58 ± 19.49 | 73.41 ± 22.97 | 0.891 |
| α-Pinene | 63.05 ± 51.72 | 4.32 ± 0.86 | 4.84 ± 0.98 | 0.973 | 44.22 ± 29.58 | 3.23 ± 1.24 | 5.77 ± 1.49 | 0.139 |
| Camphene | 4.62 ± 3.97 | 0.00 ± 0.00 | 0.00 ± 0.00 | 0.07 | 3.36 ± 2.33 | 0.00 ± 0.00 | 0.00 ± 0.00 | 0.179 |
| β-Pinene | 3.20 ± 2.57 | 1.40 ± 1.40 | 0.19 ± 0.19 | 0.491 | 2.22 ± 1.55 | 0.00 ± 0.00 | 0.59 ± 0.41 | 0.294 |
| Myrcene | 2.91 ± 2.91 | 0.00 ± 0.00 | 0.00 ± 0.00 | 0.435 | 3.88 ± 2.14 | 0.00 ± 0.00 | 0.01 ± 0.01 | 0.194 |
| *cis*-3-Hexenyl acetate | 1.83 ± 1.83 | 0.00 ± 0.00 | 0.00 ± 0.00 | 0.435 | 15.61 ± 13.55 | 16.61 ± 16.61 | 33.28 ± 20.16 | 0.392 |
| 3-Carene | 6.41 ± 3.58 | 2.48 ± 0.94 | 2.45 ± 0.69 | 0.685 | 3.24 ± 1.99 | 1.86 ± 0.97 | 3.69 ± 0.95 | 0.068 |
| Limonene | 9.38 ± 5.48 | 8.03 ± 3.39 | 6.05 ± 1.90 | 0.749 | 4.8 ± 2.79 | 6.19 ± 3.33 | 12.12 ± 4.13 | 0.067 |
| Benzyl alcohol | 13.97 ± 6.92 | 8.28 ± 1.41 | 8.42 ± 1.63 | 0.917 | 11.18 ± 3.64 | 8.10 ± 1.62 | 9.23 ± 1.67 | 0.937 |
| (*E*)-DMNT | 0.00 ± 0.00 | 0.00 ± 0.00 | 0.00 ± 0.00 | NA | 2.36 ± 1.29 | 2.59 ± 2.59 | 6.73 ± 4.69 | 0.347 |
| Methyl salicylate | 0.00 ± 0.00 | 0.00 ± 0.00 | 0.00 ± 0.00 | NA | 0.00 ± 0.00^a^ | 2.04 ± 1.49^b^ | 0.00 ± 0.00^a^ | **0.017** |
| Bornyl acetate | 1.90 ± 1.90^a^ | 4.46 ± 2.90^b^ | 0.34 ± 0.24^ac^ | **0.041** | 2.29 ± 1.59 | 1.98 ± 1.12 | 0.64 ± 0.29 | 0.556 |
| Sesquiterpene_1 | 10.44 ± 3.14 | 6.25 ± 2.75 | 4.74 ± 1.67 | 0.4 | 2.52 ± 0.92 | 1.98 ± 0.87 | 3.18 ± 0.83 | 0.521 |
| Sesquiterpene_2 | 2.62 ± 0.85 | 2.24 ± 1.37 | 2.04 ± 0.61 | 0.655 | 1.99 ± 1.21 | 2.96 ± 1.33 | 1.36 ± 0.52 | 0.586 |
| Sesquiterpene_3 | 3.52 ± 1.06 | 1.21 ± 0.94 | 2.58 ± 0.68 | 0.631 | 0.00 ± 0.00 | 0.00 ± 0.00 | 0.00 ± 0.00 | NA |
| β-Elemene | 0.52 ± 0.50 | 0.21 ± 0.21 | 0.00 ± 0.00 | 0.395 | 6.60 ± 2.99 | 3.33 ± 1.76 | 3.90 ± 1.06 | 0.435 |
| α-Longipinene | 0.00 ± 0.00 | 0.00 ± 0.00 | 0.00 ± 0.00 | NA | 2.09 ± 0.98 | 4.56 ± 3.23 | 1.91 ± 0.57 | 0.849 |
| *trans*-Caryophyllene | 0.24 ± 0.24 | 0.66 ± 0.49 | 0.74 ± 0.50 | 0.647 | 0.12 ± 0.09 | 0.46 ± 0.36 | 0.74 ± 0.49 | 0.785 |
| Aromadendrene | 0.35 ± 0.28 | 6.22 ± 4.78 | 0.13 ± 0.13 | 0.087 | 0.42 ± 0.32 | 1.08 ± 0.74 | 0.00 ± 0.00 | 0.128 |
| E,E-α-Farnesene | 0.00 ± 0.00 | 0.37 ± 0.37 | 0.04 ± 0.04 | 0.523 | 0.37 ± 0.37 | 0.66 ± 0.66 | 0.11 ± 0.11 | 0.959 |
|  |  |  |  |  |  |  |  |  |
| Total | 208.08 ± 137.75 | 67.43 ± 14.04 | 56.21 ± 12.80 | 0.741 | 57.84 ± 18.21 | 55.13 ± 20.59 | 77.05 ± 20.62 | 0.481 |
